# *Bt* rice plants may protect neighbouring non-*Bt* rice plants against the striped stem borer, *Chilo suppressalis*

**DOI:** 10.1098/rspb.2018.1283

**Published:** 2018-07-25

**Authors:** Yaoyu Jiao, Xiaoyun Hu, Yufa Peng, Kongming Wu, Jörg Romeis, Yunhe Li

**Affiliations:** 1 State Key Laboratory for Biology of Plant Diseases and Insect Pests, Institute of Plant Protection, Chinese Academy of Agricultural Sciences, No. 2 West Yuanmingyuan Road, Beijing 100193, People's Republic of China; 2 Agroscope, Research Division Agroecology and Environment, 8046 Zurich, Switzerland

**Keywords:** *Bt* rice, oviposition preference, plant volatile, intraspecific relationship, *Bt* resistance management

## Abstract

The area planted with insect-resistant genetically engineered crops expressing *Bacillus thuringiensis* (*Bt*) genes has greatly increased in many areas of the world. Given the nearby presence of non-*Bt* crops (including those planted as refuges) and non-crop habitats, pests targeted by the *Bt* trait have a choice between *Bt* and non-*Bt* crops or weeds, and their host preference may greatly affect insect management and management of pest resistance to *Bt* proteins. In this study, we examined the oviposition preference of the target pest of *Bt* rice, *Chilo suppressalis*, for *Bt* versus non-*Bt* rice plants as influenced by previous damage caused by *C. suppressalis* larvae. The results showed that *C. suppressalis* females had no oviposition preference for undamaged *Bt* or non-*Bt* plants but were repelled by conspecific-damaged plants whether *Bt* or non-*Bt*. Consequently, *C. suppressalis* egg masses were more numerous on *Bt* plants than on neighbouring non-*Bt* plants both in greenhouse and in field experiments due to the significantly greater caterpillar damage on non-*Bt* plants. We also found evidence of poorer performance of *C. suppressalis* larvae on conspecific-damaged rice plants when compared with undamaged plants. GC-MS analyses showed that larval damage induced the release of volatiles that repelled mated *C. suppressalis* females in wind tunnel experiments*.* These findings suggest that *Bt* rice could act as a dead-end trap crop for *C. suppressalis* and thereby protect adjacent non-*Bt* rice plants. The results also indicate that the oviposition behaviour of target pest females should be considered in the development of *Bt* resistance management strategies.

## 1. Introduction

Over the last 20 years, insect-resistant genetically engineered plants expressing Cry proteins from the bacterium *Bacillus thuringiensis* (*Bt*) have been rapidly and widely adopted worldwide. Introduced in 1996, they covered a total area of about 100 million hectares in 2016 [1]. In general, the adoption of *Bt* crops has provided efficient control of the target pest(s), reduced the application of pesticides and increased yields [2]. These advantages have motivated researchers in China to develop dozens of *Bt* lines for controlling lepidopteran pests of rice (*Oryza sativa* L.), including *Chilo suppressalis* (Crambidae), *Scirpophaga incertulas* (Crambidae), *Sesamia inferens* (Noctuidae) and *Cnaphalocrocis medinalis* (Crambidae) [3,4]. Among these, *C. suppressalis* is considered the most serious rice pest in China because it attacks rice at all growth stages causing an annual yield loss of 3.1% [3,5].

Although many studies have confirmed that *Bt* rice lines provide substantial protection against target pests [6], few studies have considered the effects of *Bt* rice (or *Bt* maize or cotton) on the oviposition behaviour of target pest females [7–10]. Understanding the effects of *Bt* crops on oviposition of the target pest is important because *Bt* crops are generally planted with non-*Bt* plants. The latter serve as a refuge in that they support pest reproduction and thereby reduce the selection of genotypes with resistance to *Bt* [11–13]. In the ‘refuge-in-a-bag’ approach, *Bt* and non-*Bt* seeds of a crop are mixed and sown together so that the pest females will need to travel only a short distance to select a *Bt* or non-*Bt* plant for oviposition [14]. Whether the non-*Bt* plants are in an adjacent field or are in the same field as the *Bt* plants, females of mobile target pests will have the opportunity to select between *Bt* and non-*Bt* plants for oviposition.

Most models concerning the development of *Bt* resistance in a target pest assume that oviposition among *Bt* and non-*Bt* refuge plants will be random [15,16]. That oviposition may not be random is suggested by two different views on insect behaviour. According to one view, females tend to lay eggs on plants on which their offspring can perform well. Based on this view, researchers have suspected that, given the strong selection pressure caused by *Bt* plants, female moths (most targeted pests are lepidopterans) may evolve a genetically controlled oviposition preference for alternative non-*Bt* plants [7]. A different view is based on the observation that female moths often prefer to lay eggs on undamaged rather than on insect-damaged host plants in order to reduce potential competition and improve the performance of their offspring [17]. This phenomenon had been reported for a number of lepidopteran species from different families, including *Heliothis virescens* (Noctuidae) and *Manduca quinquemaculata* (Sphingidae) on tobacco [18,19], *Ostrinia nubilalis*, *Ostrinia furnacalis* (both Crambidae) and *Spodoptera frugiperda* (Noctuidae) on corn [20–22], *Chilo partellus* (Crambidae) on African forage grass [23], *Spodoptera littoralis* (Noctuidae) on cotton [24] and *Manduca sexta* (Sphingidae) on tomato [25].

Several studies of lepidopteran oviposition preference have shown that females cannot discriminate between *Bt* and the corresponding non-*Bt* cultivars [10,26–29], suggesting that the planting of *Bt* crops will not alter adult oviposition behaviour. In these cases, however, the experiments only assessed the oviposition preference for undamaged *Bt* and non-*Bt* plants. The experiments thus ignored the potential effects of differential larval damage on *Bt* and non-*Bt* plants.

In the current study, we conducted laboratory, greenhouse and field experiments to test the hypothesis that females of the striped stem borer *C. suppressalis* (SSB) prefer to lay eggs on undamaged *Bt* rice plants over damaged non-*Bt* rice plants. If this hypothesis is correct, *Bt* plants would serve as a dead-end trap crop for the adjacent non-*Bt* plants. We also compared the performance of early-instar SSB on healthy rice plants or plants that had been damaged by conspecifics to determine whether neonate fitness differs on undamaged versus damaged rice plants. Finally, we identified the volatile organic compounds that are released by undamaged and caterpillar-damaged rice plants, which might explain the oviposition response of SBB.

## 2. Material and methods

### (a) Plants and insects

The transgenic *Bt* rice line T1C-19 and the corresponding non-transformed near isoline Minghui 63 (MH63) were used in all experiments. T1C-19 expresses a synthesized cry1C* gene driven by the maize ubiquitin promoter; the gene encodes the Cry1C protein that targets lepidopteran rice pests [30]. MH63 is an elite *indica* restorer line for cytoplasmic male sterility in China. All rice seeds were provided by Prof. Yongjun Lin (Huazhong Agricultural University, Wuhan, China).

Specimens of *C. suppressalis* used in the experiments were retrieved from a laboratory colony maintained on an artificial diet [31] for over 60 generations with annual introductions of field-collected individuals. The colony was maintained in a climate-controlled chamber (Ningbo Jiangnan, Ningbo, China) at 27 ± 3°C, 75 ± 5% RH and a photoperiod of 16 L : 8 D at the Institute of Plant Protection, Chinese Academy of Agricultural Sciences, Beijing, China.

### (b) Initial laboratory and greenhouse experiments

#### (i) Plant treatment

Pre-germinated seeds of *Bt* and non-*Bt* rice were simultaneously sown in the greenhouse at 28 ± 2°C with 65 ± 10% RH and a photoperiod of 16 L : 8 D. After 15 days, the seedlings were individually transplanted into plastic pots (diameter 20 cm, height 18 cm) with holes in the bottom and containing a 3 : 1 mixture of peat and vermiculite (Meihekou Factory, Meihekou, China). Potted plants were placed in a cement pool filled with water to a 2 cm depth. Water was replaced weekly, and nitrogenous fertilizer was applied once each week before tillering and once every two weeks after tillering. Plants were used in the experiments six weeks after transplanting when they were at the tillering stage with 10–12 leaves on the main stem.

For the caterpillar treatments, each *Bt* or non-*Bt* rice plant was individually infested with two-third instar *C. suppressalis* that had been starved for at least 2 h. The rice stems infested with caterpillars were covered with plastic sleeves to prevent insects escaping. Three days later, caterpillars had drilled into the stems and caused visible damage. Caterpillars remained in the plants for the duration of all experiments. A preliminary bioassay confirmed that third instar *C. suppressalis* could drill into the stems of *Bt* rice plants and cause damage, although the damage was significantly less on *Bt* plants than on non-*Bt* plants [32]. Plants in the treatments without damage (healthy plants) remained uninfested.

#### (ii) Dual-choice wind tunnel experiments

To test the preference of mated females of *C. suppressalis* to caterpillar-damaged or undamaged *Bt* or non-*Bt* rice plants, a dual-choice wind tunnel experiment was conducted. The wind tunnel was 230 cm long × 80 cm wide × 80 cm high (Bejing Hengfabaishun Commerce Co., Ltd, Beijing, China). The airstream in the tunnel (0.2 m s^−1^) was produced by a fan (CHW-124-401, BROAD Clean Air Technology Co. Ltd, Changsha, China) and was filtered by passage through active charcoal.

Six choice tests were conducted: (a) undamaged *Bt* rice versus undamaged non-*Bt* rice; (b) damaged non-*Bt* rice versus undamaged non-*Bt* rice; (c) damaged *Bt* rice versus undamaged *Bt* rice; (d) damaged non-*Bt* rice versus undamaged *Bt* rice; (e) damaged *Bt* rice versus undamaged non-*Bt* rice; and (f) damaged non-*Bt* rice versus damaged *Bt* rice. In each choice test, the two types of rice plants (treatments) were placed at the upwind end of the wind tunnel, 0.5 m apart. A plastic plate serving as an insect release platform was placed at the downwind end of the wind tunnel, 1.5 m away from the plants. For insect release, a cubic metal cage (226 cm^3^) containing one *C. suppressalis* female that had been mated 2 days earlier was placed on the insect release platform with an opening towards the rice plants. Each released moth was observed for 30 min, and its landing on either of the plants for at least 10 s was recorded. Moths were removed once they had made a choice. The moths that failed to make a choice within the observation period were categorized as ‘no choice’. The positions of the two rice plants were exchanged after each insect observation to eliminate a position bias. The wind tunnel was cleaned with detergent (5%, v/v) followed by alcohol (90%, v/v) and was then washed with clean water at the end of each day. Each moth was tested only once, and a total of 70–120 females were individually tested in each choice experiment. The experiments were conducted between 20.00 and 22.30 under red light (0.4 lux) conditions in a climate-controlled room at 28 ± 2°C and with 75 ± 5% RH.

#### (iii) Cage experiment in the greenhouse

In the greenhouse, four choice tests were conducted with *C. suppressalis* females: (a) undamaged *Bt* rice versus undamaged non-*Bt* rice; (b) damaged non-*Bt* rice versus undamaged non-*Bt* rice; (c) damaged *Bt* rice versus undamaged *Bt* rice; and (d) damaged non-*Bt* rice versus undamaged *Bt* rice. For each test, four potted plants (two plants of each treatment type) were positioned in the four corners of a cage (60 cm length × 60 cm width × 80 cm height) made of 80-mesh nylon nets. Plants belonging to the same treatment were positioned in opposite corners. A plastic plate containing a cotton ball saturated with a 10% honey-water solution was placed in the centre of the cage, and 10 pairs of newly emerged moths (less than 1 day) were released on the plate. After 72 h, the number of egg masses and number of eggs per egg mass on each plant were determined. Each choice test was repeated 15 to 20 times (replicates). The experiment was performed in a greenhouse at 27 ± 3°C and with 75 ± 10% RH and a photoperiod of 16 L: 8 D.

### (c) Experiments under field conditions

The oviposition preference of *C. suppressalis* females for *Bt* versus non-*Bt* rice plants was assessed in a field near Langfang City (39.5° N, 116.4° E), China. Seedlings of *Bt* and non-*Bt* rice raised as described above were transplanted into experimental plots (2 × 2 m) on 31 May 2016. Each rice line was represented by eight plots, resulting in 16 plots. Plots were arranged in a 4 × 4 grid such that the *Bt* plots alternated with the non-*Bt* plots. Plots were separated by a 1.5-m buffer. The entire experiment (16 plots) was covered with a screened cage (14 × 14 × 2.5 m) made of nylon net (3 mm mesh size) to prevent moths from entering or escaping. The plants were cultivated according to local agricultural practices but without pesticide sprays. On 29 June, 23 July and 23 August 2016, pupae and adults of *C. suppressalis* were released into the cage (300–500 individuals per release). On 30 July and 30 August 2016, the number of egg masses on each rice plant, and the number of plants damaged by caterpillars in each plot were determined. After the last determination, the number of tillers on 10 randomly selected plants in each plot was determined as an indicator of growth; two plots, one with *Bt* rice and one with non-*Bt* rice, were excluded for data analysis of egg density because of significant difference in the number of plant tillers compared to other plots (*p* < 0.05).

### (d) Caterpillar performance on damaged rice plants in a climate-controlled chamber

A bioassay was conducted to determine the fitness of *C. suppressalis* larvae that fed on non-*Bt* rice plants that were damaged or undamaged by conspecifics. Two-day-old larvae of *C. suppressalis* were weighed on an electronic balance (CPA2250, Sartorius AG, Göttingen, Germany, readability = 0.01 mg) and subsequently placed individually on plants that were undamaged or previously damaged by third instar *C. suppressalis*; as noted earlier, the instars that had previously damaged the plants were still in place. The plants were prepared as described above. After feeding for 7 days in a climate-controlled chamber (Ningbo Jiangnan, Ningbo, China) at 27 ± 3°C and 75 ± 5% RH, the surviving insects were counted and weighed. The performance of 40 insects was assessed for each of the two treatments.

### (e) Response of female *C. suppressalis* to caterpillar-induced nocturnal plant volatiles

To assess the effects of rice volatiles induced by caterpillar damage on the behaviour of *C. suppressalis* females, the nocturnal volatiles released by undamaged and caterpillar-damaged plants were collected and identified, and the responses of the females to the key induced volatiles were determined.

#### (i) Collection and analysis of rice plant volatiles

Non-*Bt* rice plants at the tillering stage and with 10–12 leaves on the main stem were used in the experiment. The plants remained undamaged or were artificially infested with third instar larvae of *C. suppressalis* (two larvae per plant) for 72 h before being used for nocturnal volatile collection (20:00–4:00). For volatile collection, the roots of the plants were washed in running water to remove soil. Two caterpillar-damaged or undamaged rice plants were then transplanted into a water-filled conical flask (250 ml) such that their roots were immersed in the water. The flask was then wrapped with aluminium foil (leaving the plant stems and leaves outside) and was placed in a glass bottle (3142 ml). The flasks were placed in a climate-controlled chamber (Ningbo Jiangnan, Ningbo, China) at 27 ± 3°C and 75 ± 5% RH. Before entering the glass bottle, air was filtered through activated charcoal, molecular sieves (5 Å, beads, 8–12 mesh, Sigma-Aldrich, St Louis, Missouri, USA) and silica gel Rubin (cobalt-free drying agent, Sigma-Aldrich, St Louis, Missouri, USA). Plant volatiles were collected in 30-mg Super Q adsorbent traps (80/100 mesh, Alltech Associates, Deerfield, IL, USA) in a glass tube (5 mm diameter, 8 cm height). Traps were rinsed with 250 ml of methylene chloride. A 500-ng quantity of nonyl acetate was added to the samples as an internal standard.

Volatiles were analysed by gas chromatography coupled with a mass spectrometry system (Shimadzu GCMS-QP 2010SE using an RTX-5 MS fused silica capillary column). Samples were injected in a 1-μl volume with a splitless injector held at 230°C. The GC-MS was operated in the scan mode with a mass range of 33–300 amu and was in an electron-impact ionization (EI) mode at 70 eV. The oven temperature was maintained at 40°C for 2 min, and was then increased 6°C min^−1^ to 250°C, where it was held for 2 min. Volatile compounds were identified by mass spectral matches to library spectra as well as by retention matches to available authentic standards. Quantifications of compounds were based on their integrated areas related to the internal standard. If standards were unavailable, tentative identifications were made based on referenced mass-spectra available from NIST (Scientific Instrument Services, Inc., Ringoes, NJ, USA) or previous study.

#### (ii) Wind tunnel experiments for identification of key repellent volatiles

No-choice olfactory tests were carried out in a wind tunnel to identify the response of mated females to 10 selected volatile compounds (electronic supplementary material, figure S1). Compounds were selected that were either significantly increased (d-limonene) or newly produced (linalool, 2-heptanol, 2-heptanone, methyl salicylate, α-pinene, α-cedrene, β-myrcene, caryophyllene and 2-nonanone) in response to damage by *C. suppressalis* larvae. In addition, one mixture containing all 10 compounds and one mixture containing a subset of five compounds (2-heptanol, α-cedrene, β-myrcene, caryophyllene and 2-nonanone) were tested based on the results for the individual compounds. The mixtures were prepared in ratios that corresponded to the ratios of compounds detected in the collection of volatiles from caterpillar-damaged rice plants. The compound 2-nonanone was purchased from Shanghai Aladdin Bio-Chem Technology Co., Ltd (Shanghai, China) with 99% purity. The remaining compounds were purchased from Sigma-Aldrich (St Louis, MO, USA) with 97–99% purity.

A rubber septum (Pherobio Technology Co., Ltd, Beijing, China) placed between two pots of undamaged rice plants at the upwind end of the flight tunnel served as the odour source. The septa were loaded with 100 µl of hexane (solvent) containing either 100 µg of an individual compound, mixtures, or no additive (control). After an adaptation period of greater than 30 min in the environment, one mated female was placed on the flight plate at the downwind end of the tunnel, and its flight behaviour was observed for 10 min. For each moth, the landing time on the plants was recorded. The moths that did not land on a plant within the observation period were categorized as ‘no response’.

### (f) Data analysis

Dual-choice wind tunnel assays were analysed with *χ*^2^ tests, with an expected response of 50% for two treatments. A paired-sample *t*-test was used to analyse the oviposition data from the greenhouse cage experiment. For the field experiment, oviposition, damage and rice tiller number were analysed by repeated measures ANOVA.

For analyses of volatiles collected from undamaged and damaged non-*Bt* plants, one-way ANOVA was used. Differences of specific compounds in two treatments were separated by Tukey honestly significant difference tests. The behavioural response of mated females to single or mixed volatiles was analysed by using the generalized linear model, with plant contact by the moth as the dependent variable and different odours as independent variables, and with a binomial distribution with the logit link function and maximum-likelihood estimation. All analyses were conducted in SPSS 22.0 (IBM SPSS, Somers, NY, USA)

## 3. Results

### (a) Preference of females for undamaged or caterpillar-damaged *Bt* or non-*Bt* rice (dual-choice wind tunnel experiment)

**Figure 1.**
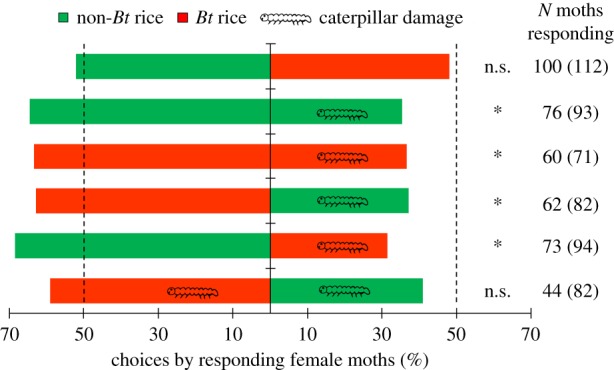
Preference of mated female *Chilo suppressalis* for undamaged or caterpillar-damaged *Bt* or non-*Bt* rice plants (dual-choice wind tunnel experiment). Caterpillar symbols indicate damage by two-third instars of *C. suppressalis.* Asterisks indicate a significant difference within a choice experiment: *p < 0.05; n.s. indicates a non-significant difference (*p* > 0.05) (*χ*^2^ test). The bars indicate the percentages of females that selected either plant type.

When given a choice between undamaged *Bt* and undamaged non-*Bt* rice plants in the wind tunnel, *C. suppressalis* females showed no preference (*χ*^2^ = 0.16, d.f. = 1, *p* = 0.689) (figure 1). However, the females exhibited a strong preference for undamaged plants over plants damaged by *C. suppressalis* larvae whether the plants were *Bt* or non-*Bt* (damaged non-*Bt* versus undamaged non-*Bt*: *χ*^2^ = 6.37, d.f. = 1, *p* = 0.012; damaged *Bt* versus undamaged *Bt*: *χ*^2^ = 4.27, d.f. = 1, *p* = 0.039; damaged non-*Bt* versus undamaged *Bt*: *χ*^2^ = 4.13, d.f. = 1, *p* = 0.042; and damaged *Bt* versus undamaged non-*Bt*: *χ*^2^ = 9.99, d.f. = 1, *p* = 0.002) (figure 1). When both *Bt* and non-*Bt* plants were damaged by caterpillars, no preference was evident (*χ*^2^ = 1.46, d.f. = 1, *p* = 0.228) (figure 1). Overall, females of *C. suppressalis* showed no preference for *Bt* versus non-*Bt* rice plants but preferred undamaged over caterpillar-damaged rice plants.

### (b) Oviposition preference of females for undamaged or caterpillar-damaged *Bt* or non-*Bt* rice (greenhouse cage experiment)

**Figure 2.**
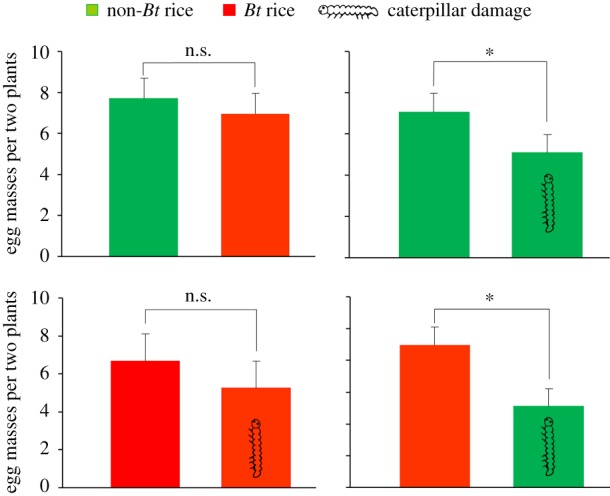
Number of egg masses laid by *Chilo suppressalis* females on undamaged or caterpillar-damaged *Bt* or non-*Bt* rice plants (greenhouse cage experiment). Caterpillar symbols indicate damage by two-third instar *C. suppressalis*. Each choice test was performed with 15 to 20 replicates, each consisting of a group of 10 pairs of *C. suppressalis* adults. The number of egg masses represents the total number laid on two rice plants. Asterisks indicate significant differences: *p < 0.05; **p < 0.01; n.s. indicates no significance (*p* > 0.05) (paired-sample *t*-test).

When given a choice between undamaged *Bt* and undamaged non-*Bt* rice in the cage experiment, *C. suppressalis* females laid a similar number of egg masses on the two types of rice plants (*t* = 1.06, d.f. = 19, *p* = 0.304) (figure 2). However, females in general laid more egg masses on undamaged over caterpillar-damaged rice plants; the difference was significant for non-*Bt* rice plants (*t* = 2.42, d.f. = 18, *p* = 0.026) but not for *Bt* rice plants (*t* = 2.03, d.f. = 14, *p* = 0.061) (figure 2). When given a choice between undamaged *Bt* rice and damaged non-*Bt* rice, females laid significantly more egg masses on the *Bt* rice plants (*t* = 3.23, d.f. = 19, *p* = 0.004). Results for the total number of eggs laid were similar to results for egg masses, i.e. the difference between damaged and undamaged *Bt* rice was again significant (*t* = 2.19, d.f. = 14, *p* = 0.046) (electronic supplementary material, figure S2). Overall, *C. suppressalis* females preferred to lay eggs on undamaged rather than on caterpillar-damaged rice plants whether the plants were *Bt* or non-*Bt*.

### (c) Egg densities of *C. suppressalis* on *Bt* and non-*Bt* rice under field conditions

**Figure 3.**
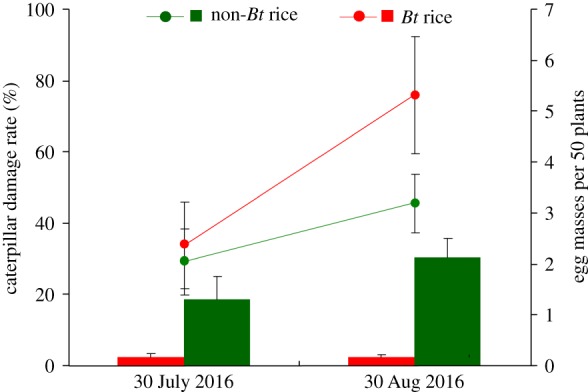
Damage rate (% of plants damaged, bars) by *Chilo suppressalis* larvae and numbers of egg masses (lines) laid by *C. suppressalis* females on *Bt* and non-*Bt* rice plants under field conditions. *Bt* and non-*Bt* rice were planted in 2 × 2 m plots in the field, and the entire plot area was covered with a screened cage (14 × 14 × 2.5 m). Pupae and adults of *C. suppressalis* were released into the cage on 29 June, 23 July and 23 August 2016. Egg masses on *Bt* and non-*Bt* rice plants were counted on 30 July and 30 August. Values are means ± s.e. The caterpillar damage rate was significantly higher (*p* < 0.001) and egg mass number was significantly lower (*p* = 0.042) on non-*Bt* plants than on *Bt* plants according to a repeated-measures ANOVA.

The caterpillar damage rates (% of plants damaged) were 18.7 ± 6.5% on 30 July 2016 and 30.3 ± 5.4% on 30 August 2016 for non-*Bt* rice plants. The damage rates were significantly lower for *Bt* rice plants than for non-*Bt* rice plants on both sampling dates (*p* < 0.001), i.e. they were less than 3% on *Bt* rice plants (figure 3). Densities of egg masses were significantly higher on *Bt* rice plants than on non-*Bt* rice plants (*F*_1,16_ = 5.29, *p* = 0.042) (figure 3).

### (d) Performance of caterpillars on damaged rice plants in a climate-controlled chamber

When 2-day-old *C. suppressalis* larvae were allowed to feed for 7 days on non-*Bt* rice plants that were either undamaged or damaged by third instars, the survival rate was significantly lower on damaged plants (75%) than on undamaged plants (92%) (*χ*^2^ = 4.50, d.f. = 1, *p* = 0.034) (electronic supplementary material, figure S3). Similarly, the increase in body weight was significantly lower on damaged plants (3.20 ± 0.22 mg) than on undamaged plants (9.29 ± 0.59 mg) (*t* = 9.19, d.f. = 65, *p* < 0.001) (electronic supplementary material, figure S3). Overall, *C. suppressalis* larvae performed much better on undamaged rice plants than on rice plants previously damaged by conspecifics.

### (e) Response of female *C. suppressalis* to caterpillar-induced nocturnal plant volatiles

A total of 28 compounds were detected in the headspace of non-*Bt* rice plants damaged by *C. suppressalis* but only four compounds (α-pinene, d-limonene, methyl salicylate and tetradecane) were detected in the headspace of undamaged non-*Bt* rice plants (electronic supplementary material, table S1). The contents of d-limonene and tetradecane were significantly higher for damaged than for undamaged plants, and the contents of α-pinene and methl salicylate did not significantly differ between damaged and undamaged plants (electronic supplementary material, table S1).

**Figure 4.**
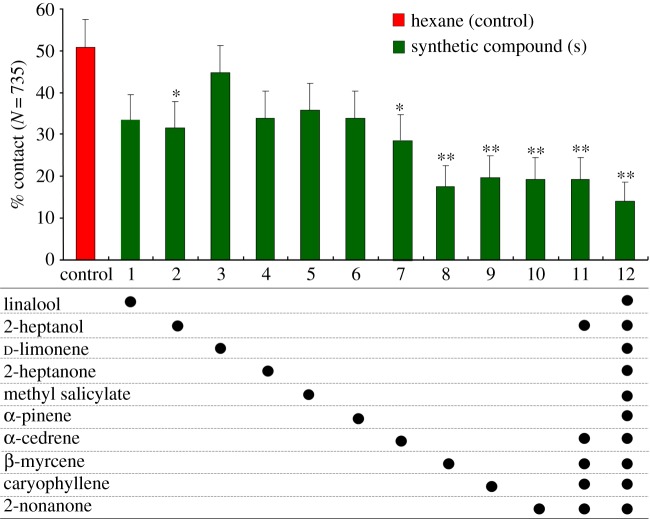
Upwind flight responses (percentage of moths that landed on plants) of *Chilo suppressalis* females to rice plants and rubber septa treated with hexane (control) or with the indicated synthetic volatile compounds dissolved in hexane (wind tunnel experiment). * and ** indicate percentages that were significantly lower (*p* < 0.05 and 0.01, respectively), i.e. indicated repellence.

Based on the GC-MS results (electronic supplementary material, table S1), we conducted a wind tunnel experiment to assess the response of mated *C. suppressalis* females to each of the following 10 volatile compounds: linalool, 2-heptanol, d-limonene, 2-heptanone, methyl salicylate, α-pinene, α-cedrene, β-myrcene, caryophyllene and 2-nonanone. In the control treatment (rice plants together with a rubber septum loaded with 100 µl of hexane), 51% of females exhibited upwind flight and contacted the rice plants (figure 4). A similar percentage of females responded to linalool, d-limonene, 2-heptanone, methyl salicylate or α-pinene (*p* > 0.05). Compared to the control treatment, significantly fewer *C. suppressalis* females showed an upwind flight response to 2-heptanol (*χ*^2^ = 3.56, d.f. = 1, *p* = 0.038), α-cedrene (*χ*^2^ = 5.74, d.f. = 1, *p* = 0.017), β-myrcene (*χ*^2^ = 11.751, d.f. = 1, *p* = 0.0003), caryophyllene (*χ*^2^ = 11.372, d.f. = 1, *p* = 0.001) or 2-nonanone (*χ*^2^ = 11.751, d.f. = 1, *p* = 0.001) (figure 4). Next, we tested the response of mated females to synthetic blends of multiple volatile compounds. The percentage of contact was only 14.3% with the blend containing all 10 compounds and was only 19.3% with the blend containing the five compounds; in both cases, the percentages were significantly lower than that of the control (both *p* < 0.001).

## 4. Discussion

The rapid increase in the planting of *Bt*-transgenic crop varieties has greatly changed crop production and protection in large areas of the world. With the combined presence of non-*Bt* crops (including those planted as refuges) and non-crop habitats, pests targeted by the *Bt* trait have a choice between *Bt* and non-*Bt* crops or weeds. The host preference of these pests has important consequences for both insect pest management and insect resistance management.

Our assays showed that *C. suppressalis* females have no oviposition preference for healthy *Bt* versus non-*Bt* rice plants. These results are consistent with previous studies with cotton, maize, rice and oilseed rape, suggesting that the transformation and the expression of the *cry* genes does not affect the oviposition behaviour of the target pests [10,26–29]. Our previous study also confirmed that undamaged *Bt* and non-*Bt* rice plants (the same rice lines used as in the current study) emitted the same number of volatiles and there were no significant differences in the quantity of each volatile compound between the treatments [33]. We did document, however, a significant oviposition preference of *C. suppressalis* females for undamaged rice plants versus plants damaged by conspecific larvae, whether the plants were *Bt* or non-*Bt*. To our knowledge, this is the first report indicating that *C. suppressalis* females avoid laying eggs on host plants damaged by conspecifics. As noted in the introduction, this phenomenon has been previously observed for a number of other lepidopteran species. However, this behaviour is not exhibited by all insects. Leaf beetles (Coleoptera: Chrysomelidae), for example, preferred damaged plants to undamaged plants [34–37]. Even some lepidopterans prefer damaged plants. *S. littoralis*, for example, preferentially oviposited on cotton plants damaged by third to fourth instar larvae rather than on undamaged plants [38]. We have also found that the mated females of another lepidopteran rice pest, *C. medinalis*, showed no oviposition preference between undamaged and conspecific-damaged rice plants (YQ Wang & YH Li 2018, unpublished data).

The question remains why *C. suppressalis* females prefer to lay eggs on healthy rice plants. According to the ‘mother knows best’ principle, one reason could be that the offspring perform better on undamaged rice plants than on damaged rice plants [17]. Our results support this hypothesis in that the survival and body weight of *C. suppressalis* larvae were lower on caterpillar-damaged rice plants than on healthy rice plants. A possible explanation is the induced production of trypsin proteinase inhibitors in rice plants in response to *C. suppressalis* infestation [39]. As has been reported for many moth species, females appear to recognize damaged plants by the volatile compounds released [18,25,40,41]. Our GC-MS analyses combined with the wind tunnel experiments indicated that five volatile compounds, namely 2-heptanol, α-cedrene, β-myrcene, caryophyllene and 2-nonanone, were mainly responsible for the repellence of damaged plants.

Based on the current finding that *C. suppressalis* females avoid laying eggs on plants previously damaged by conspecific larvae and based on the previous finding that neonate *C. suppressalis* cannot survive or cause noticeable damage on *Bt* rice plants in the field [42], we expect that *C. suppressalis* females will migrate from damaged non-*Bt* rice fields to protected *Bt* rice fields for egg laying when the *Bt* and non-*Bt* plants are in close proximity. As a consequence, the offspring produced on *Bt* rice plants will be killed by the *Bt* protein. This is inconsistent with the ‘mother knows best’ theory. As suggested by Jongsma *et al*. [7], female moths appear to be unable to detect the presence of the *Bt* proteins and may therefore fail to evolve an avoidance behaviour. Our results thus indicate that *Bt* rice could serve as a dead-end trap crop for *C. suppressalis* and would protect adjacent non-*Bt* rice as suggested by Shelton *et al*. [43]. Given the small-scale rice farming systems prevalent in China [44], the effects on *C. suppressalis* populations might be significant. This, however, needs to be confirmed under field conditions once *Bt* rice is cultivated on larger areas. Interestingly, planthoppers such as *Nilaparvata lugens* (Hemiptera: Delphacidae) show a different behaviour. When given the choice, they prefer to oviposit on caterpillar-damaged (non-*Bt*) rice plants compared to undamaged (*Bt*) rice plants [32]. Non-*Bt* rice refuges planted adjacent to *Bt* rice might thus act as a trap crop and protect *Bt* rice from this non-target pest.

Another important implication of our results relates to the evolution of *Bt* resistance in the target pests, which is a major threat for the sustainable use of *Bt* crops [12,13,45]. Currently, the most commonly practised strategy for delaying the evolution of *Bt* resistance is the establishment of refuges with non-*Bt* plants [11,12]. Because *C. suppressalis* is highly specialized for rice, structured non-*Bt* rice refuges rather than natural refuges adjacent to *Bt* rice will be necessary [16]. However, the value of the refuges in delaying the development of *Bt* resistance is based on the assumption of a random oviposition and mating by the target pest across the *Bt* plants and the non-*Bt* plants in the refuges [15,46]. Our results suggest that this assumption is invalid for *C. suppressalis* because females will avoid ovipositing on the non-*Bt* refuge plants once such plants are damaged. Data modelling for a different Lepidoptera species (*Spodoptera frugiperda*; Noctuidae) suggest that oviposition preference for *Bt* over non-*Bt* plants will significantly affect the speed of resistance evolution to the *Bt* trait [8]. This should be taken into account when designing the resistance management plan for *Bt* rice in China, especially because *C. suppressalis* can have up to five generations per year [47].

The current findings indicate that *C. suppressalis* females will show a strong oviposition preference for *Bt* over non-*Bt* rice plants once the plants have been exposed to the pest. This occurs because pest damage will be much higher on the non-*Bt* rice and because the females prefer to oviposit on undamaged plants. The results suggest that by acting as a dead-end trap crop, *Bt* rice could help protect adjacent non-*Bt* rice plants against *C. suppressalis*.

## Data accessibility

The datasets supporting this article are available from the Dryad Digital Repository: http://dx.doi.org/10.5061/dryad.nn514kg [48].
